# Longitudinal Integrated Ambulatory, Didactic, and Evidenced-Based Medicine Curriculum in Internal Medicine

**DOI:** 10.7759/cureus.45451

**Published:** 2023-09-18

**Authors:** Caroline E Moore, Elexis McBee

**Affiliations:** 1 Internal Medicine, Naval Medical Center San Diego, San Diego, USA; 2 Internal Medicine, Uniformed Services University of the Health Sciences, San Diego, USA

**Keywords:** integrated medical education, medical resident curriculum, evidence based-medicine, general internal medicine, curriculum implementation

## Abstract

Introduction: The Accreditation Council for Graduate Medical Education (ACGME) endorses evolving scholarly education regarding evidence-based medicine (EBM) and its clinical application in Internal Medicine (IM). The IM residents at Navy Medical Readiness and Training Center San Diego (NMRTC-SD) communicated the need for both increased ambulatory didactic sessions as well as a formal EBM curriculum. Prior to the academic year of 2021-2022, no formal ambulatory or EBM curriculum existed. In July 2021, an integrated EBM-ambulatory curriculum was implemented.

Methods: A pre-curriculum needs assessment was performed and thereafter an eight-session integrated ambulatory-EBM curriculum was implemented in the 2021-2022 academic year. Faculty members facilitated small group discussions focused on a particular didactic topic and EBM principle and integrated the learning of both into one session. After each session, residents completed a feedback form. At the end of the year, a post-curriculum needs assessment was collected.

Key Results: Thirty-four residents of all post-graduate years (PGY) levels participated from July 2021 to June 2022. Primary outcomes were satisfaction with the didactic portion of the curriculum, perception of receipt of adequate training in EBM principles, and perception of level of competency in practicing EBM principles. Overall, participants reported a substantial increase in satisfaction with didactic teaching and a large increase in the perceived competency in practicing EBM principles.

Conclusion: This integrated didactic-EBM curriculum represents an effective method of incorporating didactic topics in IM with the teaching and application of EBM principles, which improved resident satisfaction with the curricula and self-perception of competency in critically appraising medical literature.

## Introduction

The Accreditation Council for Graduate Medical Education (ACGME) promotes the pursuit of scholarly activity for all its governing surgical and medical subspecialties and requires evolving education for evidence-based medicine (EBM) and its application in clinical practice in Internal Medicine (IM) [1]. Additionally, it requires didactic educational experiences to advance practical knowledge in preventative care and outpatient care [1]. In IM residency programs nationwide, many have adopted independent journal clubs or workshop series aimed at guiding residents informally through the critical appraisal process. However, it is unclear if this method truly develops the skills needed to critically evaluate medical literature and its applicability to clinical practice. Additionally, there is significant variation between residency programs in their objectives, teaching structure and experience, participation, and meeting frequency [2]. There have been no specific, standardized processes for conducting a successful journal club published, but the literature suggests that regular meetings, trained leaders to lead the discussion, and the use of standardized appraisal tools aid in success [3]. EBM is critical for Internists to integrate the best research evidence available with patient-centric values and clinical expertise. As the advancement of medicine rapidly progresses with a subsequent increase in the volume (and access) of literature, it has never been more important for physicians to develop the skills to critically appraise literature and learn to practice EBM, which begins with learning fundamentals. The ACGME describes a responsibility to ensure trainees can approach patient care in an evidence-based manner [1], and thus a more standardized method is required among IM residency programs.

Many barriers exist to developing and executing a successful EBM curriculum; barriers that have been identified, although not all-inclusive, are time restraints, faculty indifference, standardization, lack of trained faculty, and lack of awareness of the full scope of EBM resources available [4]. It has also been theorized that incorporating EBM training into ongoing didactic curriculums within a residency program may offer a more complete teaching model compared to separate, independent journal clubs [4]. This theory may also be more optimal with respect to the adult learning theory where a resident may be able to immediately apply the EBM principles being taught directly to the didactic topic.

At our institution, Navy Medical Readiness and Training Center San Diego (NMRTC-SD), similar to the national landscape of EBM teaching in IM, a standardized EBM training model had not been implemented with significant variability among residents in their exposure to formal EBM teaching. It was identified that the approach to formal education and training of EBM concepts could be optimized to best enable residents to “develop appropriate clinical questions, efficiently search the medical literature, and use standardized methods to assess the quality of medical evidence” to guide their medical decision-making [1]. Based on this identification, a pre-curriculum needs assessment capturing all IM residents was performed and the results reiterated the need for increased EBM training as well as a formal, ongoing ambulatory curriculum with structured didactic topics. Specifically, the assessment highlighted a lack of volume of didactic sessions, dissatisfaction with the EBM and ambulatory curriculums, and decreased perception of competence and adequate training in EBM principles. Given the lack of data surrounding best practices on a formal EBM curriculum, as well as a desire to fit educational sessions within typical working hours, a curriculum was developed to meet the residents’ needs and to occur within a four-hour protected time slot on Friday afternoons during the ambulatory week (of a traditional 3+1 rotation model). A curriculum during this time would offer a significant opportunity to increase the utilization of protected educational time internally within the NMRTC-SD IM program to enrich resident education while meeting ACGME milestones, maximizing time and resources already available, and increasing education of EBM principles.

To address the resident needs identified during the needs assessment, a longitudinal, integrated EBM-ambulatory curriculum was created for all IM residents. Each session of the curriculum was designed to focus on one didactic topic (i.e., hypertension) in conjunction with education on a pertinent EBM principle that would maximize the utilization and efficiency of academic time and offer recurring practice to improve the likelihood of retention of EBM skills [5]. After extensive literature review, this curriculum appears to represent the first integrated curriculum of its kind the literature designed for small group teaching and discussion. The primary goals for the curriculum were to (1) increase the consistency of academic sessions, (2) increase residents’ perception of competence in EBM principles, and (3) increase the perception of adequate training in ambulatory didactics and EBM principles. Secondary goals included (1) assessment of the best teaching methods of academic sessions, and (2) addressing strengths and weaknesses of the installed integrated curriculum.

## Materials and methods

Overview

An integrated ambulatory-EBM curriculum for the IM program was developed in the spring of 2021 and implemented at the start of the academic year in July 2021 to optimize protected didactic time and offer a robust educational experience to meet resident and academic needs.

**Figure 1:**
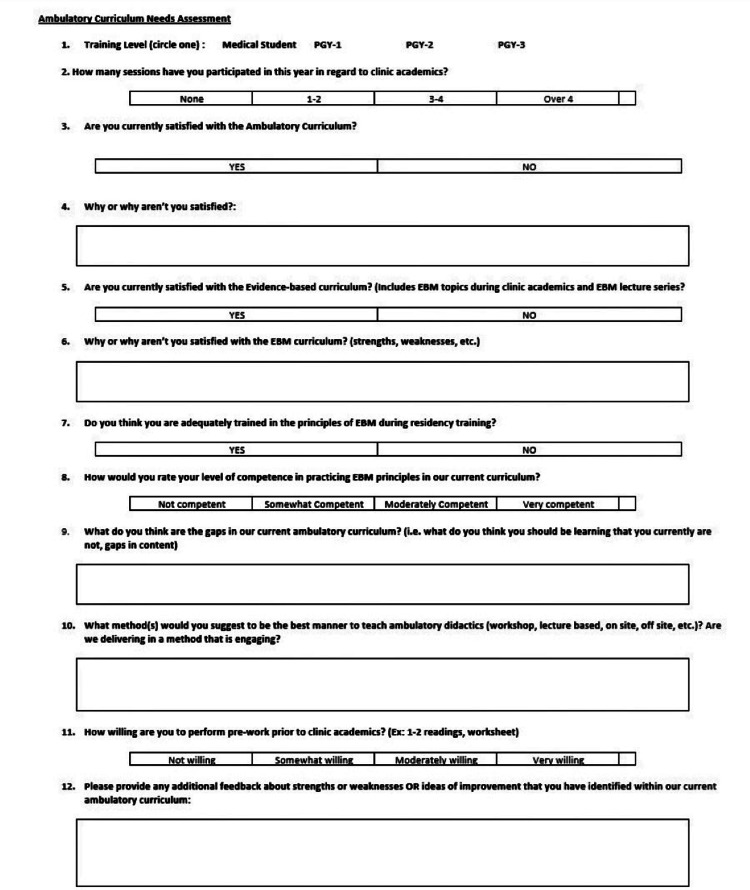
Pre- and Post-Curriculum Needs Assessment

A targeted needs assessment was created and distributed in spring 2021 to all IM residents (PGY1 through PGY3) (Appendices, Figure 1). This data was collected and analyzed to develop an integrated, structured curriculum that combined the education of core didactic topics in IM with important EBM principles essential for progressive autonomy. The curriculum was developed as an eight-session curriculum per academic year with the thought that it could be expanded over a two-year timeframe to capture many didactic topics that occur in IM. The topics were selected based on core topics of IM for clinical practice and board exams, recently published and available literature, and a review of the existing curriculum. The sessions occurred on Friday afternoons over a four-hour time period. IM faculty members as well as several sub-specialty faculty members, volunteered for sessions. The educational objectives for the curriculum and each individual session were posted to faculty members prior to the start of the academic year (Tables 1, 2).

**Table 1:** Curriculum Goals and Objectives CEX - Clinical Evaluation Exercise

Objective	Educational Method	ACGME Core Competency Addressed	Evaluation Method
Ambulatory Specific			
Apply and demonstrate competent knowledge and skill related to the presentation, evaluation, and management of chronic and acute illnesses encountered in the primary care setting	1 - Small group facilitator sessions, workshops, and/or lecture series on core clinical concepts	Medical Knowledge Patient Care	Direct Observation MultiSource Evaluations Annual In-Training Exam before vs. after CEX
Demonstrate knowledge of health maintenance guidelines and standard of care	1 - Small group facilitator sessions, workshops, and/or lecture series on core clinical concepts	Medical Knowledge Patient Care	Direct Observation MultiSource Evaluations Annual In-Training Exam before vs. after
To prepare residents for autonomous practice at the GMO and IM Staff level by demonstrating an understanding of the skills critical to the practice of outpatient medicine	1 - Small group facilitator sessions, workshops, and/or lecture series on core clinical concepts	Medical Knowledge Patient Care	Direct Observation MultiSource Evaluations Annual In-Training Exam before vs. after CEX
Describe and explain the basic principles for interpreting diagnostic tests, including cost-effectiveness, high-value care	1 - Small group facilitator sessions, workshops, and/or lecture series on core clinical concepts	Medical Knowledge Patient Care	Direct Observation MultiSource Evaluations Annual In-Training Exam before vs. after
EBM Specific			
Residents will demonstrate the ability to develop a PICO question and use it effectively	Cohort Journal Club Sessions	Practice-Based Learning and Improvement	Satisfactorily participate in EBM sessions EBM presentation
Residents will demonstrate knowledge of various study designs and statistical techniques	Cohort Journal Club Sessions, Lecture and Reading on Introduction to Medical Statistics	Practice-Based Learning and Improvement Medical Knowledge	Taylor EBM questionnaire Satisfactorily participate in EBM sessions EBM presentation Observed Critical Appraisal by Staff
Increase residents’ confidence in their ability to critically appraise and apply medical literature	Cohort Journal Club Sessions	Practice-Based Learning and Improvement	Taylor EBM questionnaire Satisfactorily participate in EBM sessions EBM presentation CEQ Questionnaire
Residents completing the EBM curriculum will demonstrate use of the principles directly in application to patient care	Cohort Journal Club Sessions	Practice-Based Learning and Improvement	Satisfactorily participate in EBM sessions EBM presentation Observed Critical Appraisal by Staff

**Table 2:** Individual Session Learning Objectives RCT - randomized controlled trial, RR - relative risk, NNT - number needed to treat, NNH - number needed to harm, PICO - population, intervention, comparison, outcomes, HIV - human immunodeficiency virus, PrEP - pre-exposure prophylaxis, POLST - Physician Orders for Life-Sustaining Treatment

Lesson	Learning/Educational Format	Resources	Objectives
1. Introduction to Evidence-Based Medicine: Study Design and Biostatistics with Critical Appraisal Tool Overview	Lecture (i.e., Nutrition Conference Room) Time: 120 minutes	1-2 Staff Lecturers for 4 separate weeks. Audiovisual Equipment: Computer with Projector capabilities	1. Compare and contrast various study designs to include Cohort, RCT, Meta-Analyses, Cohort Studies, and Descriptive studies be able to describe the strengths, limitations, and study goals/use, 2. Calculate and describe sensitivity, specificity, RRR, RR, ARR, NNT/NNH, PPV, NPV, likelihood ratios, Odds ratio. 3. Define and compare statistical significance and clinical significance and interpret p-values and confidence intervals. 4. Identify and describe biases of various study designs and how it limits data interpretation. 5. Demonstrate ability to work through critical appraisal tool.
2a. Formulating a Clinical Question and Performing a Literature Search. 2b. Hypertension	Workshop Time: 120 minutes Lecture/Workshop Time: 90 minutes	1 (or 2) Staff Audiovisual Equipment: Computer with Projector capabilities, 1 computer between residents (could potentially ask to bring laptops/personal computers) or use library Staff prepare lecture/workshop/small group discussion that address objectives	1. Define evidence-based medicine (EBM) 2. Execute the steps of the EBM process. 3. Examine a clinical case and identify the foreground question and the associated study needed to answer that question. 4. Develop and ask clinical foreground questions using PICO. 5. Demonstrate ability of Literature search via PubMed. 6. Cite the definition(s) and differentiate essential and secondary hypertension. 7. Define the components of an initial evaluation for a patient with newly diagnosed hypertension. 8. Describe the guideline treatment goals for hypertension and the rationale (via chosen article). 9. Describe the classes of antihypertensives and their indications for use as well as most common side effects with a focus on assessing efficacy and medication titration. 10. Recognize white coat hypertension, masked hypertension, hypertensive urgency, and emergency
3a. Appraisal of Articles about Therapy or Prevention 3b. Type 2 Diabetes Mellitus	Small Group (Cohort) Discussion Time: 90 minutes Lecture/Workshop Time: 90 minutes	1 Staff (acting as Facilitator). Both Residents and facilitator will review the article in advance. Facilitators should be prepared to guide discussion and residents should be prepared to actively participate. No specific equipment is necessary. Staff prepare lecture/workshop/small group discussion that address objectives	1. Describe the components of the study design in a RCT to include randomization process, inclusion/exclusion criteria, and designation of study outcomes. 2. Interpret Hazard Ratios and Kaplan-Meier curves. 3. Calculate the NNT and NNH and interpret the clinical applicability. 4. List and explain potential biases within the paper. 5. Apply McMaster’s criteria (in worksheet) to determine clinical applicability. 6. Define the diagnostic criteria for Type 2 Diabetes Mellitus and the approach to labeling an appropriate HbA1c goal based on life expectancy and comorbidities. 7. Describe the classes of available pharmacologic treatment options to include oral antiglycemics, insulin, and injectable non-insulin regimens, which classes are preferred and reasoning. 8. Review cost effective treatment choices for individuals living in the United States. 9. Be able to provide nutritional education to patients.
4a. Appraisal of Articles about Diagnosis 4b. HIV	Small Group (Cohort) Discussion Time: 90 minutes Lecture/Workshop Time: 90 minutes	1 Staff (acting as Facilitator). Both Residents and facilitator will review the article in advance. Facilitators should be prepared to guide discussion and residents should be prepared to actively participate. No specific equipment is necessary. Staff prepare lecture/ workshop/small group discussion that address objectives	1. Describe Likelihood ratios and their use. 2. Calculate PPV & NPV and how it would apply to patient care (i.e., how to use the test). 3. Calculate Sensitivity and specificity. 4. Compare and contrast linear and logistic regression. 5. Apply McMaster’s criteria (in worksheet) to determine clinical applicability. 6. Describe the symptoms of acute retroviral syndrome and clinical stages of HIV infection. 7. Explain the diagnostic process and algorithm in diagnosed HIV and interpret the results. 8. Define the indications for PrEP. 9. Understand the surveilling features required for follow up and long-term complication risks (i.e., cardiovascular disease)
7a. How to Use Guidelines and Recommendations about Screening 7b. Update on Cancer Screening Guidelines	Small Group (Cohort) Discussion Time: 90 minutes Lecture/Workshop Time: 90 minutes	1 Staff (acting as Facilitator). Both Residents and facilitator will review the article in advance. Facilitators should be prepared to guide discussion and residents should be prepared to actively participate. No specific equipment is necessary. Staff prepare lecture/workshop/small group discussion that address objectives	1. Use published criteria to critically analyze screening recommendations. 2. Explore the benefits and harms of screening and calculate ARR and RRR to aid in measurement of benefit. 3. Apply sensitivity and specificity to screening modalities. 4. Define the biases that may be present with identifying early diagnoses. 5. Develop an approach on how to discuss screening with patients based on individual risk factors. 6. Explain how to manage abnormal results and when it is appropriate to refer to Oncology. 7. Review and understand the most up-to-date guidelines on breast, lung, colon, prostate, cervical, and skin cancers. 8. Consider High Value Care decisions when consider screening tests.
8a. Appraisal of Meta-Analyses 8b. Palliative Care	Small Group (Cohort) Discussion Time: 90 minutes Lecture/Workshop Time: 90 minutes	1 Staff (acting as Facilitator). Both Residents and facilitator will review the article in advance. Facilitators should be prepared to guide discussion and residents should be prepared to actively participate. No specific equipment is necessary. Staff prepare lecture/workshop/small group discussion that address objectives	1. Define meta-analyses and describe its uses. 2. Explain heterogeneity and its role in meta-analyses. 3. Define reporting bias (and other biases) and identify in meta-analyses. 4. Use validated guidelines for analyzing meta-analysis to interpret its use in your patient population. 5. Explain the differences between palliative and hospice care and which patient populations are eligible and can derive benefit from these services. 6. Describe where palliative and hospice care can occur (home vs hospital, etc.). 7. Explore and discuss the culture-specific needs of patients and families as they approach End-Of-Life (EOL) care that reflects our patient populations cultural diversity. 8. Practice approaching a Goals of care discussion with a patient and key points to highlight and understand institutional advance directive and POLST components. 9. Discuss the treatment for common EOL symptoms (pain, constipation, nausea)
9. PGY3 Demonstration of Critical Appraisal Skills	Cohort Workshop/Discussion led by PGY3’s Time: 120-150 minutes (pending # of PGY3’s in selected Cohort)	1 Staff for Observation of facilitation by Senior Resident PGY3s choose a recent article (within 6 months) and distribute to Cohort; They will lead a small group on the critical appraisal	1. Apply principles of EBM directly to a recently published article. 2. Demonstrate ability to identify a clinical question, preferably from a clinic patient using PICO format and the accurate study design preferred. 3. Lead a small group confidently in discussion on pertinent methods, statistical analysis and calculations, stance on statistical and clinical significance

Execution

The eight-session curriculum was developed and didactic topics and learning objectives were jointly formulated by Residency leadership (Chief Resident, Program Director, and Assistant Program Director(s)), based upon the results of the needs assessment, the educational objectives of the IM Program, and the ACGME guidelines (Table 2) [1]. EBM principles were adapted from JAMA’s “User’s Guide to Medical Literature” [6-11].

Curricular sessions occurred in various places throughout the year (at the discretion of the facilitator). This included conference rooms on campus as well as off-campus sites (i.e., coffee shops, parks) to promote wellness and participation. The curriculum was built to promote the participation of residents in the context of small-group teaching to increase discussion. Sessions generally lasted between 3.5 hours. Small-group cohorts included a variable number of residents at all levels of training (up to 8-9 individuals from PGY1 through PGY3). Participation did not affect the residents’ clinical evaluations.

**Figure 2:**
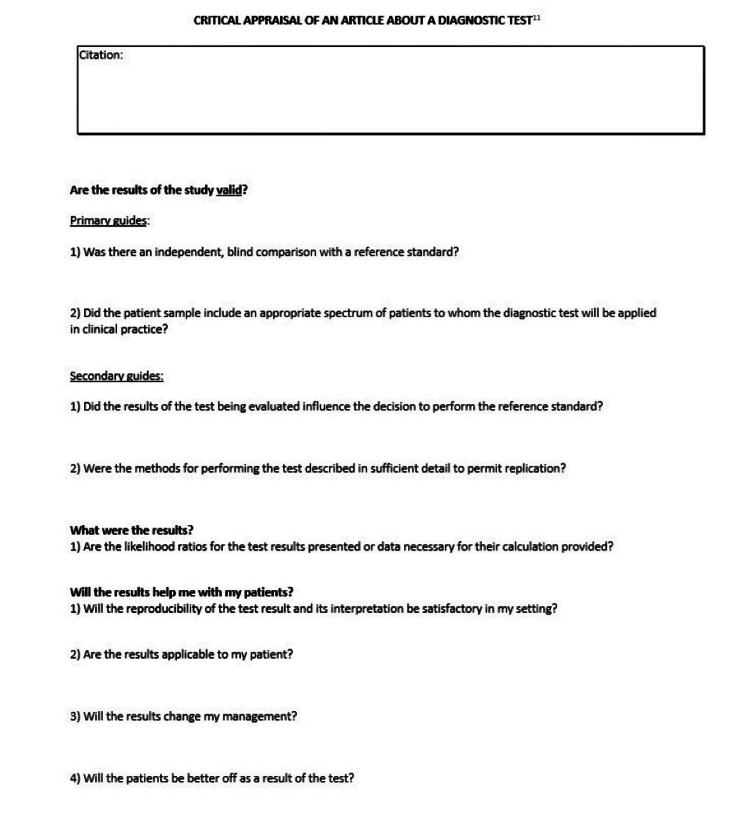
Example Critical Appraisal Tool

One week prior to the scheduled curricular session, residents in the small-group session were introduced to the session-specific curricular objectives, given the pre-reading literature for the EBM portion of the discussion, a critical appraisal worksheet tool (if applicable) (Appendices, Figure 2), other curricular content if applicable, and reminded three days prior of the upcoming session via email by the Chief Resident. During the curricular session, the core clinical topic was discussed (i.e., diabetes) as well as a specific EBM principle (i.e., appraisal of articles about therapy or prevention) which was reflected in the chosen scientific literature article(s) discussed during that session. The eighth session of the curriculum was designed to allow demonstration of the learned EBM principles by senior residents as they took turns leading the small group through the critical appraisal process of a scientific article.

Evaluation and feedback

**Figure 3:**
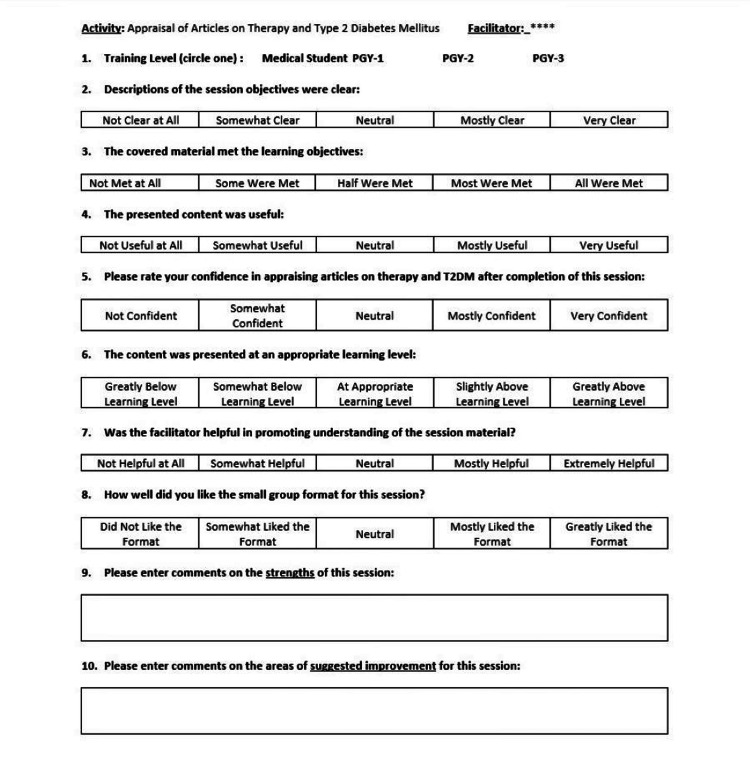
Example of Session Feedback Form

Following each session, residents were given a feedback form for the session (standardized across all sessions) to assess residents’ perception of the effectiveness of the session and to evaluate for future program improvement. A 10-question survey feedback form (example in Appendices, Figure 3) was developed for this purpose. This survey form was designed to assess constructs related to the usefulness of the curriculum and residents’ confidence in their abilities to critically appraise the literature. In addition, the form also allowed for open-ended comments regarding the strengths and areas of improvement for each session. The form was designed using a 5-point Likert scale and adapted from the Course Experience Questionnaire (CEQ) [12].

At the end of the academic year, a post-curriculum needs assessment was distributed electronically to all IM residents in the program to assess their satisfaction with the EBM curriculum and perceptions of competency. This post-curriculum needs assessment mirrored the pre-curriculum needs assessment. The primary outcomes included satisfaction with the didactic portion of the curriculum, resident perception of adequacy of training in principles of EBM, and perception of self-competency in practicing EBM principles (from not competent to very competent). Secondary outcomes that were assessed included the number of sessions attended by residents, satisfaction with the evidence-based medicine teaching, themes from qualitative remarks regarding strengths, and weaknesses, and why residents either were or were not satisfied with the curriculum. Preferred modes of learning were also queried. The results were compared to the pre-curriculum needs assessment and shared with Program Leadership to review and address.

Data from the pre- and post-assessment was analyzed using a mixed-method approach. Descriptive statistics were generated for quantitative survey items. Qualitative data from open-ended survey items was analyzed using opening coding that searched for emerging themes.

## Results

Thirty-four IM residents of all PGYs participated in the longitudinal, integrated didactic, and EBM curriculum during the academic year 2021-2022 and data was collected during this time period only. Average attendance was six residents per session over the course of the academic year (residents are only allowed to take vacation during outpatient rotations and some residents were absent due to vacation). Twenty-one residents participated in the pre-curriculum needs assessment and 27 residents participated in the post-curriculum needs assessment (response rate of 79%). There was an even distribution by PGY level of residents answering the pre- and post-curriculum needs assessments.

For the primary outcomes, satisfaction with the didactic portion of the curriculum increased from 4.8% to 92.6% after curriculum implementation (Table 3). Additionally, the pre-curriculum needs assessment noted that only 23.8% of residents perceived that they were adequately trained in EBM principles. This increased to 88.9% following curriculum implementation. In the pre-curriculum needs assessment, the majority of residents reported that they were “somewhat competent” (61.9%) in practicing EBM principles with only 4.8% reporting “very competent”. After curriculum implementation, the majority of residents reported being “moderately competent” (70.4%) with 11.1% perceiving themselves as “very competent” (Table 3). Results for the secondary outcomes showed that prior to the initiation of the integrated curriculum, 57.1% of residents attended only 1-2 structured academic sessions that may or may not have included EBM teaching; however, after curriculum implementation, the majority of residents (59.3%) attended four or more structured didactic and EBM teaching sessions (with only 7.4% attending 1-2 sessions). Residents reported an increase in the satisfaction of EBM teaching, from 38.1% to 88.9% (Table 3).

**Table 3:** Comparison of Post-Targeted Needs Assessment to Pre-Targeted Needs Assessment Pre-curricular assessment results within paratheses. EBM - evidence-based medicine, PGY - post-graduate year

Statement	Question Set (A)			
Question Set (A)	PGY1	PGY2	PGY3	
What is your PGY level?	33.3% (33.3%)	29.6% (33.3%)	37% (33.3%)	
Question Set (B)	>4	3-4 sessions	1-2 sessions	None
How many sessions have you participated in this year in regard to clinic academics?	59.3% (0%)	33.3% (23.8%)	7.4% (57.1%)	0% (0%)
Question Set (C)	Yes	No		
Are you currently satisfied with the ambulatory curriculum?	92.6% (4.8%)	7.4% (95.2%)		
Are you currently satisfied with the EBM curriculum?	88.9% (38.1%)	11.1% (61.9%)		
Do you think you are adequately trained in the principles of EBM during your residency training?	88.9% (23.8%)	11.1% (76.2%)		
Question Set (D)	Very competent	Moderately Competent	Somewhat Competent	Not competent
How would you rate your level of competence in practicing EBM principles in our current curriculum?	11.1% (4.8%)	70.4% (14.3%)	18.5% (61.9%)	0% (19%)

The results described in Table 4 depict average scores for the session-specific survey forms collected following each session across all modules of the academic year. Thematically, the average scores illustrate that across all of the modules, the residents perceived that the session objectives were made clear, the covered content was useful and met the described learning objectives, and the majority of residents were confident in the material after the session (Table 4). Furthermore, residents perceived that the content was presented at an appropriate learning level and largely enjoyed the format of the sessions (Table 4). Individual session feedback survey results can be found in Tables 5-12.

**Table 4:** Average Scoring across All Modules (8) and Learning Sessions Other questions included in this survey were qualitative answers on strengths and weaknesses of this particular session.

Average Scoring across All Modules (8) and Learning SessionsStatement	Strongly Agree (%)	Agree (%)	Neutral (%)	Disagree (%)	Strongly Disagree (%)
Descriptions of the session learning objectives were clear.	78.1%	18.3%	1.9%	1.8%	0.0%
The covered material met the learning objectives.	83.4%	14.9%	0.8%	0.9%	0.0%
The presented content was useful.	77.5%	19.0%	2.2%	1.3%	0.0%
I am confident in the session-specific topic* after this session.	52.0%	39.1%	7.1%	1.7%	0.0%
The content was presented at an appropriate learning level.	73.3%	13.7%	1.9%	4.8%	6.3%
The facilitator was helpful in promoting understanding of the session material.	79.1%	16.0%	4.0%	0.9%	0.0%
I enjoyed the format of this session.	69.1%	23.1%	5.6%	1.3%	0.9%

**Table 5:** Module 1 Survey Results: Intro to Evidence-Based Medicine: Study Design and Biostatistics with Critical Appraisal Tool Overview

Statement	Strongly Agree (%)	Agree (%)	Neutral (%)	Disagree (%)	Strongly Disagree (%)
Descriptions of the session were clear.	46.4%	35.7%	3.6%	14.3%	0.0%
The covered material met the learning objectives.	57.1%	35.7%	0.0%	7.1%	0.0%
The presented content was useful.	25.0%	53.6%	10.7%	10.7%	0.0%
I am confident in Biostatistics, Study Design, and formulating a clinical question after this session.	3.6%	67.9%	21.4%	7.1%	0.0%
The content was presented at an appropriate learning level.	3.6%	92.6%	0.0%	3.6%	0.0%
The facilitator was helpful in promoting understanding of the session material.	25.0%	42.9%	25.0%	7.1%	0.0%
I enjoyed the format of this session.	3.6%	57.1%	21.4%	10.7%	7.1%

**Table 6:** Module 2 Survey Results: Formulating a Clinical Question/Hypertension

Statement	Strongly Agree (%)	Agree (%)	Neutral (%)	Disagree (%)	Strongly Disagree (%)
Descriptions of the session were clear.	75.0%	25.7%	0.0%	0.0%	0.0%
The covered material met the learning objectives.	78.6%	21.4%	0.0%	0.0%	0.0%
The presented content was useful.	78.6%	21.4%	0.0%	0.0%	0.0%
I am confident in formulating a clinical question and Hypertension after this session.	53.6%	42.9%	3.6%	0.0%	0.0%
The content was presented at an appropriate learning level.	78.6%	7.1%	0.0%	14.3%	0.0%
The facilitator was helpful in promoting understanding of the session material.	89.3%	10.7%	0.0%	0.0%	0.0%
I enjoyed the format of this session.	64.3%	28.6%	7.1%	0.0%	0.0%

**Table 7:** Module 3 Survey Results: Appraisal of Screening Guidelines/Updates on Cancer Screening

Statement	Strongly Agree (%)	Agree (%)	Neutral (%)	Disagree (%)	Strongly Disagree (%)
Descriptions of the session were clear.	84.2%	15.8%	0.0%	0.0%	0.0%
The covered material met the learning objectives.	89.5%	10.5%	0.0%	0.0%	0.0%
The presented content was useful.	89.5%	10.5%	0.0%	0.0%	0.0%
I am confident in appraising screening guidelines and updates on cancer screening after this session.	57.9%	36.8%	5.3%	0.0%	0.0%
The content was presented at an appropriate learning level.	84.2%	5.3%	10.5%	0.0%	0.0%
The facilitator was helpful in promoting understanding of the session material.	94.7%	5.3%	0.0%	0.0%	0.0%
I enjoyed the format of this session.	89.5%	5.3%	5.3%	0.0%	0.0%

**Table 8:** Module 4 Survey Results: Appraisal of Articles on Therapy/Type 2 Diabetes Mellitus T2DM - Type 2 Diabetes Mellitus

Statement	Strongly Agree (%)	Agree (%)	Neutral (%)	Disagree (%)	Strongly Disagree (%)
Descriptions of the session were clear.	95.2%	4.8%	0.0%	0.0%	0.0%
The covered material met the learning objectives.	95.2%	4.8%	0.0%	0.0%	0.0%
The presented content was useful.	100.0%	0.0%	0.0%	0.0%	0.0%
I am confident in appraising articles on therapy and T2DM after this session.	81.0%	19.0%	0.0%	0.0%	0.0%
The content was presented at an appropriate learning level.	95.2%	0.0%	0.0%	4.8%	0.0%
The facilitator was helpful in promoting understanding of the session material.	100.0%	0.0%	0.0%	0.0%	0.0%
I enjoyed the format of this session.	100.0%	0.0%	0.0%	0.0%	0.0%

**Table 9:** Module 5 Survey Results: Disclosure of Medical Errors (In-Person Simulation)

Statement	Strongly Agree (%)	Agree (%)	Neutral (%)	Disagree (%)	Strongly Disagree (%)
Descriptions of the session were clear.	78.3%	21.7%	0.0%	0.0%	0.0%
The covered material met the learning objectives.	87.0%	13.0%	0.0%	0.0%	0.0%
The presented content was useful.	73.9%	26.1%	0.0%	0.0%	0.0%
I am confident in disclosing medical errors in an interprofessional setting after this session.	47.8%	43.5%	8.7%	0.0%	0.0%
The content was presented at an appropriate learning level.	91.3%	4.3%	4.3%	0.0%	0.0%
The facilitator was helpful in promoting understanding of the session material.	82.6%	17.4%	0.0%	0.0%	0.0%
I enjoyed the format of this session.	56.5%	39.1%	4.3%	0.0%	0.0%

**Table 10:** Module 6 Survey Results: Conducting Family Meetings in Palliative Care

Statement	Strongly Agree (%)	Agree (%)	Neutral (%)	Disagree (%)	Strongly Disagree (%)
Descriptions of the session were clear.	60.0%	33.3%	6.7%	0.0%	0.0%
The covered material met the learning objectives.	73.3%	20.0%	6.7%	0.0%	0.0%
The presented content was useful.	66.7%	26.7%	6.7%	0.0%	0.0%
I am confident in conducting a family meeting after this session.	33.3%	46.7%	13.3%	6.7%	0.0%
The content was presented at an appropriate learning level.	80.0%	0.0%	0.0%	6.7%	13.3%
The facilitator was helpful in promoting understanding of the session material.	60.0%	33.3%	6.7%	0.0%	0.0%
I enjoyed the format of this session.	66.7%	26.7%	6.7%	0.0%	0.0%

**Table 11:** Module 7 Survey Results: Appraising Articles of a Diagnostic Test and HIV HIV - human immunodeficiency virus

Statement	Strongly Agree (%)	Agree (%)	Neutral (%)	Disagree (%)	Strongly Disagree (%)
Descriptions of the session were clear.	85.7%	9.5%	4.8%	0.0%	0.0%
The covered material met the learning objectives.	95.2%	4.8%	0.0%	0.0%	0.0%
The presented content was useful.	95.2%	4.8%	0.0%	0.0%	0.0%
I am confident in appraising articles on a diagnostic test and HIV after this session.	57.1%	38.1%	4.8%	0.0%	0.0%
The content was presented at an appropriate learning level.	81.0%	0.0%	0.0%	0.0%	19.0%
The facilitator was helpful in promoting understanding of the session material.	90.5%	9.5%	0.0%	0.0%	0.0%
I enjoyed the format of this session.	90.5%	9.5%	0.0%	0.0%	0.0%

**Table 12:** Module 8 Survey Results: Senior Resident Led Journal Club

Statement	Strongly Agree (%)	Agree (%)	Neutral (%)	Disagree (%)	Strongly Disagree (%)
Descriptions of the session were clear.	100.0%	0.0%	0.0%	0.0%	0.0%
The covered material met the learning objectives.	90.9%	9.1%	0.0%	0.0%	0.0%
The presented content was useful.	90.9%	9.1%	0.0%	0.0%	0.0%
I am confident in participating in a journal club after this session.	81.8%	18.2%	0.0%	0.0%	0.0%
The content was presented at an appropriate learning level.	72.7%	0.0%	0.0%	9.1%	18.2%
The facilitator was helpful in promoting understanding of the session material.	90.9%	9.1%	0.0%	0.0%	0.0%
I enjoyed the format of this session.	81.8%	18.2%	0.0%	0.0%	0.0%

Additionally, qualitative remarks were collected and analyzed and revealed four recurring themes: (1) Benefit of the workshop format for learning for learning, (2) Well-being benefit associated with teaching at offsite locations, (3) Satisfaction with the integrated curricular format, (4) Importance of topic choice for teaching sessions.

(1) Residents endorsed the benefit of workshop-based learning and appreciated that the sessions were not in lecture format. For example:

“Group workshops like we have been doing are also conducive to enduring participation and maintaining engagement. I do not feel lectures would be very effective for the topics we have covered so far in the curriculum.”

“Reviewing papers is good. Not lectures.”

(2) Residents appreciated the opportunity to participate in academic sessions at off-site locations removed from patient care. It appears that this improved well-being. For example:

“Off-site is excellent and conducive to creating a low-stress environment in which to learn these topics.”

“Important topics covered in a structured yet casual setting in a way that is done to get us out of the hospital.”

(3) Residents were enthusiastic about the integrated teaching format and expressed a desire for this teaching format to be used in the future. For example:

“I think this was a huge step in the right direction for our education.”

“Do something similar next year please!”

(4) Residents endorsed that the choice of topics for teaching sessions to include topics that are high-yield and vary from one academic year to the next is useful and desired. For example:

“Huge improvement from last year with high yield topics with EBM incorporated.”

“Continue the curriculum next year to cover other topics common in primary care.”

“Relevant clinical information that was useful and delivered in a good block of time.”

## Discussion

This longitudinal, integrated didactic and EBM curriculum in our Internal Medicine residency program is an effective method of incorporating both the ACGME’s didactic and evidence-based medicine requirements [13]. The results show that our curriculum not only improved resident satisfaction with the didactic and EBM teaching portions of the curriculum, but it also increased residents’ perception of training adequacy in EBM principles and perceived self-competency in using EBM in clinical practice. Furthermore, the volume of didactic and EBM sessions improved dramatically which was a gap identified in the pre-curriculum needs assessment. Thematically, it was observed from qualitative feedback that residents were very receptive to the chosen topics and were eager to expand topics the following year, valued off-campus locations for academic sessions, and generally were satisfied with the new curriculum. Specifically, it was commented that off-site locations created a low-stress environment that was helpful for participation compared to having the sessions taught on campus. Thus, our curriculum allowed for standardized, scheduled monthly meetings within typical working hours in small groups at off-site locations that allowed for optimal participation. Overall, our results demonstrated success in achieving large improvements in the primary and secondary outcomes, answering the targeted needs identified by the IM residents, and improving the perception of EBM competency in line with ACGME requirements. This integrated curricular structure offers an efficient and effective model to teach EBM principles.

Advantages of this curriculum include regularly scheduled teaching sessions within typical working hours that are without time constraints and can be achieved within a four-hour time window, which is a common barrier identified in executing a successful EBM curriculum [4]. Additionally, each session is standardized to have a similar format based on standardized critical appraisal tools [6-11] and the didactic and EBM portions of the session directly complement each other to facilitate learning. For example, if the chosen didactic topic is type 2 diabetes mellitus and the EBM topic is “Appraisal of Articles on Therapy”, all residents receive the same pre-reading literature that illustrates this particular EBM topic. During the session, the didactic learning objectives for diabetes mellitus are discussed and then the focus transitions to critically appraising medical literature centered around therapy for diabetes. For additional standardization, the same faculty member ideally would teach this session across all cohorts to ensure every cohort has a similar learning experience.

This curriculum does have some limitations. The data only represents one academic center and its applicability to other centers is unclear. However, the content and teaching methods are applicable across institutions and specialties. In addition, not all residents who participated completed the pre-needs and post-needs assessments which may have affected our results. Additionally, due to residency program scheduling constraints, Friday afternoons were the only available time slot for the curriculum to occur on a consistent basis. In the future, the curriculum may be of greater success with increased participation if performed on another day during the week. Due to our residents being active-duty military members, we were unable to plan or conduct sessions during July/June (the beginning and the end of the academic year, respectively) due to planned military moves to alternative bases in and out of the country, which altered the number of sessions that could be taught in one academic year. If this curriculum were to be implemented at a different institution or program type, a consideration for additional topics per academic year could be made. Finally, not all residents completed the pre- and post-curriculum needs assessment which may have marginally affected the resulting data.

This curriculum introduces an integrated didactic-ambulatory and EBM curriculum and a standardized process to incorporate the teaching of EBM principles in a structured format. This format is noted to be favorable as it may offer a more complete teaching model [4]. The results demonstrate the impact of this integrated curriculum over just a one-year period, significantly improving resident satisfaction with the didactic teaching, increasing the perception of the adequacy of training and competency in practicing EBM principles in medicine and increasing overall satisfaction with the curriculum. Given the structure of this integrated curriculum, it could easily be implemented not just in IM residency programs but could be adapted to any medical or surgical subspecialty. Future studies could further assess this curriculum as an intervention and analyze the long-term impacts of training and implications on clinical practice.

## Conclusions

In conclusion, this curriculum introduces an integrated didactic-ambulatory and EBM curriculum and a standardized process to incorporate the teaching of EBM principles in a structured format. It represents an effective method of incorporating both didactics topics in IM with teaching and application of EBM principles, and this format is noted to be favorable as it may offer a more complete teaching model. The results demonstrate the impact of this integrated curriculum over just a one-year period, significantly improving resident satisfaction of the didactic teaching, increasing perception of the adequacy of training and competency in practicing EBM principles and critically appraising medical literature, and increasing overall satisfaction with the curriculum. Given the structure of this integrated curriculum, it could easily be implemented not just in IM residency programs but could be adapted to any medical or surgical subspecialty. Integrated curriculums may be one of the best methods to meet evolving didactic and evidence-based medicine requirements. Future studies could further assess this curriculum as an intervention and analyze the long-term impacts of training, performance evaluations (i.e. test scores), and implications on clinical practice.

## References

[1] Accreditation Council for Graduate Medical Education (2021). ACGME Program Requirements for Graduate Medical Education in Internal Medicine. Published.

[2] Gottlieb M, King A, Byyny R, Parsons M, Bailitz J (2018). Journal club in Residency Education: an evidence-based guide to best practices from the Council of Emergency Medicine Residency Directors. West J Emerg Med.

[3] Deenadayalan Y, Grimmer-Somers K, Prior M, Kumar S (2008). How to run an effective journal club: a systematic review. J Eval Clin Pract.

[4] Carpenter CR, Kane BG, Carter M, Lucas R, Wilbur LG, Graffeo CS (2010). Incorporating evidence-based medicine into resident education: a CORD survey of faculty and resident expectations. Acad Emerg Med.

[5] Ilic D, Tepper K, Misso M (2012). Teaching evidence-based medicine literature searching skills to medical students during the clinical years: a randomized controlled trial. J Med Libr Assoc.

[6] Murad MH, Montori VM, Ioannidis JP (2014). How to read a systematic review and meta-analysis and apply the results to patient care: users' guides to the medical literature. JAMA.

[7] Agoritsas T, Merglen A, Shah ND, O'Donnell M, Guyatt GH (2017). Adjusted analyses in studies addressing therapy and harm: users’ guides to the medical literature. JAMA.

[8] Birch DW, Eady A, Robertson D, De Pauw S, Tandan V (2003). Users' guide to the surgical literature: how to perform a literature search. Can J Surg.

[9] Guyatt GH, Sackett DL, Cook DJ (1993). Users' guides to the medical literature. II. How to use an article about therapy or prevention. A. Are the results of the study valid? Evidence-Based Medicine Working Group. JAMA.

[10] Guyatt GH, Sackett DL, Cook DJ (1994). Users' guides to the medical literature. II. How to use an article about therapy or prevention. B. What were the results, and will they help me in caring for my patients? Evidence-Based Medicine Working Group. JAMA.

[11] Jaeschke R, Guyatt G, Sackett DL (1994). Users' guides to the medical literature. III. How to use an article about a diagnostic test. A. Are the results of the study valid? Evidence-Based Medicine Working Group. JAMA.

[12] Steele G, West S, Simeon D (2003). Using a modified course experience questionnaire (CEQ) to evaluate the innovative teaching of medical communication skills. Educ Health (Abingdon).

[13] Edgar L, Fig LM (2012). ACGME milestone project. J Nucl Med.

